# Exploring Hematological Parameters and Their Prognostic Value in Adult COVID-19 Patients: Insights from Mogadishu, Somalia

**DOI:** 10.1155/2023/8862457

**Published:** 2023-10-03

**Authors:** Abdirasak Sharif Ali Mude, Abd Elhadi Mohamed Agena Musa

**Affiliations:** ^1^Faculty of Medicine and Health Sciences, Simad University, Mogadishu, Somalia 252; ^2^Faculty of Medical Laboratory Sciences, Elimam Elmahdi University, Kosti, Sudan 249

## Abstract

There were no data on SARS-CoV-2 and hematology in Mogadishu, Somalia, despite the fact that many prior investigations of SARS-CoV-2 and hematology have already been conducted in many different parts of the world. As a result, this study aimed to assess hematological changes in COVID-19-infected patients at some selected hospitals in Mogadishu, Somalia. *Methods*. Outright, 433 COVID-19 patients were included in this study, which used a hospital-based cross-sectional design to investigate hematological alterations using the Mindray full automated hematological analyzer. Furthermore, ethical considerations were taken into account during the study. All individuals provided informed consent prior to participation in the study. Data were analyzed using SPSS. *Results*. The median age of the current study was 54.65 ± 20.486 years. People with diabetes, high blood pressure, asthma, or heart disease made up 21.2%, 21.2%, 20%, and 2.1% of the study population, respectively. According to the patients' hematological profiles, 89.5% of them had leukopenia, 86.8% had lymphopenia, and 89% had neutrophilia. Monocytes, eosinophils, basophils, and thrombocytes were typically normal although around 50.4% individuals exhibited anemia. *Conclusion*. Hematological indicators can predict how bad the illness is and how it will turn out, which helps guide clinical therapy. Leukopenia, neutrophilia, lymphopenia, and anemia were found in this study. At the time of admission, a thorough review of laboratory parameters can help clinicians make a treatment plan and quickly give intensive care to the patients who need it most.

## 1. Introduction

Since December 2019, a global pandemic has been caused by the coronavirus disease 2019 (COVID-19)-infected pneumonia [1, 2]. Globally, 13,150,645 patients were infected as of July 15, 2020, with 574,464 deaths confirmed by WHO (Novel Coronavirus) [3]. COVID-19's natural reservoir and intermediate host have yet to be described, but laboratory evidence of infection with a related coronavirus has been found in several wildlife species, including bats and pangolins [4]. Emerging data suggest that COVID-19 should be viewed as a systemic disease involving multiple systems, including the cardiovascular, respiratory, gastrointestinal, neurological, hematopoietic, and immune systems. Although it is well known that COVID-19 primarily manifests as a respiratory tract infection [5], the studies on patients with COVID-19 have found some variations in their hematologic and immunologic indices. It was discovered that the disease's acute stage was characterized by significant lymphopenia and a sharp loss of CD4+ T and CD8+ T cells. The relationship between anomalies in laboratory indices such as leukocyte, lymphocyte, and eosinophil counts, serum inflammatory cytokine levels, and the severity or mortality of the diseases has been examined in several research studies on SARS-CoV, 11 MERS-CoV, 12, or SARS-CoV-213 infections [6].

There were no data on SARS-CoV-2 and hematology in Mogadishu, Somalia, despite the fact that many prior investigations of SARS-CoV-2 and hematology have already been conducted in many different parts of the world. To the best of our knowledge, no particular research on SARS-CoV-2 and hematology has been conducted in Mogadishu, Somalia. As a result, this study aimed to assess hematological changes in COVID-19-infected patients at some selected hospitals in Mogadishu, Somalia. The research can provide context-specific information, contribute to scientific knowledge, and potentially inform clinical practice in the management of COVID-19 in similar settings. This study will allow for a localized investigation of hematological parameters in COVID-19 patients, taking into account the unique population characteristics and resource limitations.

## 2. Materials and Methods

### 2.1. Study Design and Populations

A hospital-based cross-sectional study was carried out at some selected hospitals in Mogadishu, Somalia, to assess hematological parameters among COVID-19 patients. The study is undertaken between December 1st, 2020, and March 30th, 2021. Patients experiencing respiratory symptoms are chosen to take part in this study. Approximately 433 inpatients and outpatients were selected for the study after excluding anyone who denied participation.

### 2.2. Data Collection Tools and Procedures

An already-structured questionnaire was used to obtain information from patients experiencing respiratory problems. Trained health professionals in some selected hospitals collected sociodemographic factors and COVID-19-related clinical data. There was strict confidentiality about the information provided on the questionnaires, and the data were double-checked daily to ensure accuracy.

### 2.3. Sample Collection

About 5 ml of blood samples were drawn from study participants using an EDTA (ethylenediaminetetraacetic acid, containing vacutainer tube) for analysis of hematological parameters.

## 3. Investigation Using Mindray BC-2800 Automated Hematological Analyzer

### 3.1. Technical Specifications

#### 3.1.1. Parameters

The parameters include WBC, Lymph#, Mid#, Gran#, Lymph%, Mid%, Gran%, RBC, HGB, HCT, MCV, MCHC, RDW-CV, RDW-SD, PLT, MPV, PDW, PCT, and Histogram for WBC, RBC, and PLT.

#### 3.1.2. Principles

An electrical impedance method for counting and a cyanide-free method for hemoglobin were used in this study.

#### 3.1.3. Performance

The performance is given in Table1.

#### 3.1.4. Sample Volume

The sample volume is as follows: prediluted 20 *μ*L and whole blood 13 *μ*L.

#### 3.1.5. Aperture Diameter

The aperture diameter is 80 *μ*m.

#### 3.1.6. Throughput

Throughput is 30 samples per hour.

### 3.2. Data Analysis

The study findings were analyzed using version 25.0 of IBM's SPSS statistical analysis program.

### 3.3. Ethics Approval and Informed Consent

Research Ethics Subcommittee of the University of Simad's School of Medicine and Health Sciences approved the study, and all patients enrolled did so voluntarily and after receiving appropriate information about the study. The approval conditions included a need that the research be carried out in compliance with local and applicable laws.

## 4. Results

### 4.1. Sociodemographic Characteristics of Study Participants

In the present study, a total of 433 people participated, 57.3% of whom were male and 42.7% female. The average age of participants was 54.65 years old, with a standard deviation of 20.486. Most of the participants (83.4%) were married, with the rest (16.6%) being single. Of the participants, 76.2% were educated and possessed a high school diploma, while 23.8% were not educated. Employment-wise, 78.5% of the participants were employed and 21.5% were unemployed. Out of the participants, 91.7% were not smokers and 8.3% were smokers (Table 2).

### 4.2. Comorbidities Status of Study Participants

Diabetic, hypertensive, asthmatic, and cardiac comorbidities accounted for 21.2%, 21.2%, 20%, and 2.1% (Table 3).

### 4.3. Hematological Characteristics of Study Participants

This study provided unique insights into the numerous blood parameters of COVID-19 patients, both severe and nonsevere. Let us examine and expand these findings: the study found that patients with severe COVID-19 had significantly lower amounts of RBCs, Hb, and Hct than those with nonsevere instances. A decrease in these parameters, which are important components of the blood that are crucial for oxygen transport and overall health, could indicate anemia or decreased oxygen-carrying capacity. This finding may indicate the severity of the impact of the disease on the general physiological function of the body. The study also discovered that the majority of patients' mean cell volume, which measures the size of red blood cells, was within the normal range. This shows that sickness had little effect on the size of red blood cells. This part of the data could imply that despite the severity of COVID-19 infection, certain characteristics of the red blood cell population remained relatively steady. In individuals with severe COVID-19, leukopenia (lower white blood cell count) and lymphocytopenia (lower count of a specific type of white blood cell, called lymphocytes) were particularly common. These findings indicate that the body's immune response is disrupted, potentially leaving patients exposed to infections due to a reduced defense system. This could be related to the severity of the disease and its impact on the ability of the immune system to combat infections. It is interesting to note that patients with severe COVID-19 exhibit a large increase in neutrophils, a different type of white blood cell. A vigorous inflammatory response in the body, possibly as a result of the immune system's attempt to fight the infection, can be indicated by neutrophilia. However, severe inflammation can also cause tissue damage, which may increase disease severity. Other types of white blood cells, such as monocytes, eosinophils, and basophils, were within the normal range in individuals with severe COVID-19. This could imply that the severity of sickness had no effect on the specific components of the immune system response. Interestingly, the majority of COVID-19 patients had normal thrombocyte (platelet) numbers. Platelets are essential for blood clotting and wound healing. This finding implies that patients with severe COVID-19 did not consistently demonstrate abnormalities in platelet counts. However, even with normal platelet counts, it is important to investigate whether platelet function is impaired (Table 4).

### 4.4. Regression Analysis

COVID-19 infection rates were similar in the study groups with low or high hemoglobin, erythrocyte, hematocrit, and mean cell volume than those with normal levels (OR = 0.686, 95% CI 0.339–1.388, *>*0.05; OR = 1.27, 95% CI 0.590–2.738, *>*0.05; OR = 0.786, 95% CI 0.363–1.701, *>*0.05; OR = 0.913, 95% CI 0.403–2.069, *p* > 0.05, respectively). Neutrophil and monocyte count among study individuals were associated with an increased risk of COVID-19 infection (OR = 0.175, 95% CI 0.43–0.714, *<*0.05; OR = 0.94, 95% CI 0.050–0.755, *<*0.05, respectively). Study participants with abnormally low or high levels of leukocytes, lymphocytes, and eosinophils were no more likely to contract COVID-19 than those with normal levels (OR = 1.115, 95% CI 0.564–2.207, *>*0.05; OR = 0.000, 95% CI 0.000–1.2, *>*0.05; OR = 1.336, 95% CI 0.456–3.91, *>*0.05, respectively). Participants in the study who had low basophil and thrombocyte counts were 0.062 and 0.250 times more likely to obtain COVID-19 infection in comparison to those with normal parameters (OR = 0.062, 95% CI 0.024–0.163, *<*0.05; OR = 0.250, 95% CI 0.089–0.701, *<*0.05, respectively) (Table 5).

### 4.5. Hematological Parameters That Can Predict the Severity of COVID-19 Infection

In Table 6, hemoglobin levels are revealed, and the distribution of anemic cases shows that 63.0% of severe/critical patients (SCPs) and 63.6% of nonsevere/critical patients (NSCPs) fall within this category. Likewise, the distribution of polycythemia cases comprises 15.9% of SCP and 16.1% of NSCP, while the normal category encompasses 21.0% of SCP and 20.3% of NSCP. These results suggest that there is no significant difference in hemoglobin levels between SCP and NSCP. Regarding erythrocyte levels, 50.1% of SCP and 50.4% of NSCP exhibit low counts, while erythrocytosis is observed in 30.5% of SCP and 30.6% of NSCP. Normal erythrocyte counts are found in 19.4% of SCP and 19.0% of NSCP. Similarly, there is no significant difference in erythrocyte levels between SCP and NSCP. Analysis of hematocrit levels reveals that 62.6% of SCP and 63.4% of NSCP fall into the low category, while high hematocrit is observed in 18.7% of SCP and 18.2% of NSCP. Normal hematocrit levels are present in 18.7% of SCP and 18.4% of NSCP. Hematocrit levels also do not show a significant difference between SCP and NSCP.

With regard to leukocyte counts, 28.6% of SCP and 89.5% of NSCP exhibit leukopenia, while leukocytosis is seen in 7.4% of SCP and 90.6% of NSCP. Normal leukocyte counts are found in 63.9% of SCP and 88% of NSCP. Interestingly, there is a notable difference in leukocyte levels. SCP patients are more likely to have leukopenia, while NSCP patients are more likely to have leukocytosis. Concerning neutrophil levels, neutropenia is observed in 2.5% of SCP and 63.6% of NSCP, while neutrophilia is noted in 74.6% of SCP and 89% of NSCP. Normal neutrophil counts are present in 22.9% of SCP and 90.9% of NSCP. Neutrophil levels also show a significant difference. SCP patients are more likely to have neutrophilia, while NSCP patients are more likely to have neutropenia. Regarding lymphocyte levels, lymphopenia is prevalent in 78.8% of SCP and 86.8% of NSCP, while lymphocytosis is observed in 8.8% of SCP and 92.1% of NSCP. Normal lymphocyte counts are found in 12.5% of SCP and 88.9% of NSCP. Lymphocyte levels show a significant difference. SCP patients are more likely to have lymphopenia, while NSCP patients are more likely to have lymphocytosis.

## 5. Discussion

The current study involved a total of 433 participants, with 57.3% males and 42.7% females. The mean age of the participants was 54.65 ± 20.486 years. The majority of the participants were married (83.4%), while 16.6% were single. In terms of education, 76.2% of the participants were educated, while 23.8% were uneducated. Regarding employment status, 78.5% of the participants were employed, while 21.5% were unemployed. The majority of the participants were nonsmokers (91.7%), while 8.3% were smokers (see Table 2). According to these findings, the study participants were mostly middle-aged and married, with a higher number of males than females. The high proportion of educated participants indicates that the sample was diversified and had a good representation of various educational backgrounds. It is worth noting that a sizable proportion of the individuals was working, implying a potential link between employment status and the health outcome under investigation. Furthermore, the high proportion of nonsmokers in the sample could be attributed to growing public awareness of the negative health effects of smoking. The study was carried out by the authors in [7]. The mean age was lower than our study, and the COVID-19 patients who were admitted was 48.798 ± 8.53 years. Another study was carried out by the authors in [8]. The median age of 201 patients was 51 years, and 128 (63.7% of the total) were men. In addition to another study [9], a total of 52 patients were included in the study, with a mean age of 59.7 (standard deviation of 13.3) years and a prevalence of men of 67%.

The outcomes of this study provide crucial insights into the comorbidities present in COVID-19 patients. According to the findings, a large proportion of COVID-19 patients had preexisting medical disorders, with diabetes and hypertension accounting for 21.2% each and asthmatic patients accounting for 20%. It should be noted that the prevalence of cardiac comorbidities was rather low, at only 2.1% (see Table 3). These findings emphasize the importance of understanding the underlying health status of COVID-19 patients. Diabetes and hypertension are two preexisting illnesses that may enhance the risk of severe sickness and consequences from COVID-19. Therefore, minimizing the effects of COVID-19 on these patients may require early detection and management of comorbidities. The authors in [7] discovered that diabetic, hypertensive, asthmatic, and cardiac comorbidities accounted for 68%, 73%, 35%, and 3%, respectively. In addition [10], they discovered diabetes (12.50%), hypertension (20.59%), asthma (8.09%), and cardiac issue (30.88%). The study carried out by the authors in [11] identified hypertension (19% versus 23.2%), diabetes (9% versus 10.9%), chronic obstructive pulmonary disease (COPD) (3% versus 8.6%), and cardiovascular disease (6% versus 1.8%). In a study conducted by [12], the 95% confidence intervals for hypertension, diabetes, cardiovascular, cerebrovascular, and respiratory diseases in patients with severe cases were 2.79 (95% CI: 1.66–4.69), 1.64 (95% CI: 2.30−1.08), 1.79 (95% CI: 1.08–2.96), 3.92 (95% CI: 2.45–6.28), and 1.98 (95% CI: 1.26–3.12), respectively. Another study carried out by the authors in [9] found that 91 (48%) patients had a comorbidity, with coronary heart disease (15 (8%) patients), diabetes (36 (19%) patients, and hypertension (58 (30%) patients) being the most prevalent.

The erythrocytes, hemoglobin, and hematocrit levels were lower in severe COVID-19 patients than in nonsevere COVID-19 patients (63.6%, 50.4%, and 63.4%, respectively), but the mean cell volume was normal in the vast majority of patients (73.8%). Leukopenia and lymphocytopenia were seen in 89.5% and 86.8%, respectively, in the severe COVID-19 patients. Neutrophilia, on the other hand, was considerably greater in patients with severe COVID-19 (89%). In the severe COVID-19 patients, monocytes (94.9%), eosinophils (88.7%), and basophils (91.55%) were all within normal norms. In 87.3% of the study, participants had normal thrombocyte levels. The findings of this study show that there are substantial disparities in hematological parameters between COVID-19 patients who are severe and those who are not. Severe COVID-19 patients, in particular, had lower red blood cell, hemoglobin, and hemocrit levels than nonsevere patients. This shows that severe COVID-19 cases may be associated with anemia, which may contribute to the disease's severity. Furthermore, the study discovered that leukopenia and lymphocytopenia were more common in severe COVID-19 patients, indicating a possible immune system compromise. Neutrophilia, on the other hand, was much greater in patients with severe COVID-19, which could be attributed to the body's immune response to the viral infection. The normal ranges of monocytes, eosinophils, basophils, and thrombocyte counts indicate that these measures may not have a substantial impact on COVID-19 severity. According to a study by [13], COVID-19 individuals exhibit thrombocytopenia (36.2%) and lymphopenia (83.2%); this study disagrees our study which found normal platelet in the majority of the patients. In addition, the study done by the authors in [14] discovered that the majority of patients had a normal CBC upon admission (normal Hb, WBC, and platelet count). In addition, none of the patients displayed moderate or severe thrombocytopenia, which is typically seen in other viral diseases like the prevalent dengue fever in our area. However, lymphopenia was seen in 28% of all patients. According to the study carried out by the authors in [15], majority of the patients had significantly higher neutrophil (NEU) counts (*p*=0.0001) and lower white blood cell (WBC) counts (*p*=0.0001).

A limitation of our study is the use of only some hematological parameters, such as the complete blood count. In addition, the number of samples collected was limited due to a relative lack of funds and the short duration of data collection. The generalizability of the findings may be limited due to the sample's geographical restriction to Mogadishu, which may not fully represent the entire pregnant population in Somalia. In order to facilitate future investigations, we suggest conducting a complete blood count test followed by a confirmatory test or employing an advanced technique such as molecular techniques to understand the exact effect of SARS-CoV-2 on blood cells and coagulation parameters.

In conclusion, hematological indicators can predict how bad the illness is and how it will turn out, which helps guide clinical therapy. Leukopenia, neutrophilia, lymphopenia, and anemia were found in this study. At the time of admission, a thorough review of laboratory parameters can help clinicians make a treatment plan and quickly give intensive care to the patients who need it most.

## Figures and Tables

**Table 1:** Technical specifications using Mindray BC-2800 automated hematology analyzer.

Parameters	Linearity	Precision (CV%)
WBC (10⁹/L)	Range	3 (4.0–15.0)
RBC (10^12^/L)	0.0–99.9	2 (3.0–6.5)
HGB (g/L)	0.0–9.99	2 (100–180)
MCV (fL)	0–300	1 (70–100)
PLT (10⁹/L)	0–999	5 (200–500)

**Table 2:** Sociodemographic characteristics of study participants.

Sociodemographic characteristics	*N* %	COVID-19 status	
		SCP	NSCP
*Age*			
20–39	133 (30.7)	15 (11.3)	118 (88.7)
40–59	62 (14.3)	54 (87.1)	8 (12.9)
60–79	199 (46.0)	179 (89.9)	20 (10.1)
>80	39 (9.0)	34 (87.2)	5 (12.8)

*Gender*			
Male	248 (57.3)	240 (96.8)	8 (3.2)
Female	185 (42.7)	145 (78.4)	40 (21.6)

*Marital status*			
Single	72 (16.6)	67 (93.1)	5 (6.9)
Married	361 (83.4)	318 (88.1)	43 (11.9)

*Education*			
No education	103 (23.8)	79 (76.7)	24 (23.3)
Educated	330 (76.2)	306 (92.7)	24 (7.3)

*Occupation*			
Employed	340 (78.5)	315 (92.6)	25 (7.4)
Unemployed	93 (21.5)	70 (75.3)	23 (24.7)

*History of smoking*			
Yes	36 (8.3)	33 (91.7)	3 (8.3)
No	397 (91.7)	352 (88.7)	45 (11.3)

SCP: severe COVID-19 patient; NSCP: none severe COVID-19 patient.

**Table 3:** Comorbidities status of study participants.

Comorbidities	*N* %	COVID-19 status		
		SCP	NSCP	
*Diabetes mellitus*				
Yes	92 (21.2)	78 (84.8)	14 (15.2)	
No	341 (78.8)	34 (10.0)	307 (90.0)	

*Hypertension*				
Yes	92 (21.2)	80 (86.9)	12 (13.0)	
No	341 (78.8)	34 (10.0)	307 (90.0)	

*Asthma*				
Yes	20 (4.6)	19 (95.0)	1 (5.0)	
No	413 (95.4)	47 (11.4)	366 (88.6)	

*Heart disease*				
Yes	9 (2.1)	9 (100.0)	0 (0.0)	
No	424 (97.9)	48 (11.3)	376 (88.7)	

**Table 4:** Hematological characteristics of study participants.

Parameters	*N* %	COVID-19 status	
		SCP	NSCP
*Hemoglobin*			
Anemic	273 (63.0)	245 (63.6)	28 (58.3)
Polycythemia	69 (15.9)	62 (16.1)	7 (10.0)
Normal	91 (21.0)	78 (20.3)	13 (27.1)

*Erythrocyte*			
Low	217 (50.1)	194 (50.4)	23 (47.9)
Erythrocytosis	132 (30.5)	118 (30.6)	14 (29.2)
Normal	84 (19.4)	73 (19.0)	11 (22.9)

*Hematocrit*			
Low	271 (62.6)	244 (63.4)	27 (56.3)
High	81 (18.7)	70 (18.2)	11 (22.9)
Normal	81 (18.7)	71 (18.4)	10 (20.8)

*Mean cell volume*			
Microcytic	69 (15.9)	61 (15.8)	8 (16.7)
Macrocytic	46 (10.6)	40 (10.4)	6 (12.5)
Normocytic	318 (73.4)	284 (73.8)	34 (70.8)

*Leukocyte*			
Leukopenia	124 (28.6)	111 (89.5)	13 (10.5)
Leukocytosis	32 (7.4)	29 (90.6)	3 (9.4)
Normal	277 (63.9)	245 (88)	32 (11.6)

*Neutrophil*			
Neutropenia	11 (2.5)	7 (63.6)	4 (36.4)
Neutrophilia	323 (74.6)	288 (89)	35 (10.8)
Normal	99 (22.9)	90 (90.9)	9 (9)

*Lymphocyte*			
Lymphopenia	341 (78.8)	296 (86.8)	45 (13.2)
Lymphocytosis	38 (8.8)	35 (92.1)	3 (7.9)
Normal	54 (12.5)	48 (88.9)	6 (11.1)

*Monocyte*			
Monocytopenia	14 (3.2)	11 (78.6)	3 (21.4_
Monocytosis	57 (13.2)	30 (52.6)	27 (50.9)
Normal	359 (82.9)	341 (94.9)	18 (5)

*Eosinophil*			
Eosinopenia	46 (10.6)	42 (91.3)	4 (8.7)
Eosinophilia	15 (3.5)	1386.7)	2 (13.3)
Normal	372 (85.9)	330 (88.7)	42 (11.3)

*Basophil*			
Basopenia	20 (4.6)	8 (40)	12 (60)
Basophilia	24 (5.5)	21 (95.8)	3 (12.5)
Normal	389 (89.8)	356 (91.5)	33 (8.5)

*Thrombocyte*			
Thrombocytopenia	18 (4.2)	12 (66.7)	6 (33.3)
Thrombocytosis	37 (8.5)	37 (100)	0 (0)
Normal	378 (87.3)	336 (88.9)	42 (11.1)

**Table 5:** Regression analysis of hematological parameters among COVID-19 patients.

Parameters	B	S.E.	Wald	Sig	Exp (B)	95% CI for exp (B)	
						Lower	Upper
*Hemoglobin*							
Lowe Hb	−0.071	0.480	0.022	0.882	0.931	0.363	2.387
High Hb	−0.344	0.532	0.417	0.518	0.709	0.250	2.011

*Red blood cell*							
Low RBC	0.326	0.470	0.481	0.488	1.386	0.551	3.484
High RBC	1.033	0.617	2.804	0.094	2.810	0.839	9.413

*Hematocrit*							
Low Hct	−0.926	0.509	3.308	0.069	0.396	0.146	1.075
High Hct	−1.070	0.498	4.617	0.032	0.343	0.129	0.910

*Mean cell volume*							
Low MCV	−0.091	0.418	0.048	0.827	0.913	0.403	2.069
High MCV	−0.225	0.474	0.226	0.634	0.798	0.315	2.021

*Neutrophil*							
Low neutrophil	−1.289	0.999	1.664	0.197	0.275	0.039	1.954
High neutrophil	0.190	0.494	0.147	0.701	1.209	0.459	3.186

*Lymphocyte*							
Low lymphocyte	−1.841	0.541	11.579	0.001	0.159	0.055	0.458
High lymphocyte	0.372	0.359	1.069	0.301	1.450	0.717	2.933

*Monocyte*							
Low monocyte	−1.642	0.695	5.587	0.018	0.194	0.050	0.755
High monocyte	−2.836	0.359	62.42	0.000	0.059	0.029	0.119

*Eosinophil*							
Low eosinophil	2.414	1.178	4.199	0.040	11.184	1.111	112.9
High eosinophil	1.493	1.287	1.345	0.246	4.450	0.357	55.4

*Basophil*							
Low basophil	2.33	1.234	4.992	0.030	10.178	0.112	110.5
High basophil	1.232	1.112	1.267	0.270	4.652	0.267	54.3

*Platelets*							
Low platelets	1.277	0.738	2.997	0.083	3.587	0.845	15.233
High platelets	1.342	0.822	3.123	0.092	3.722	0.934	15.822

**Table 6:** Hematological parameters that can predict the severity of COVID-19 infection.

Parameters	*N* %	COVID-19 status	
		SCP	NSCP
*Hemoglobin*			
Anemic	273 (63.1%)	245 (63.6%)	28 (58.3%)
Polycythemia	69 (15.9%)	62 (16.1%)	7 (10.0%)
Normal	91 (21.0%)	78 (20.3%)	13 (27.1%)

*Erythrocyte*			
Low	217 (50.1%)	194 (50.4%)	23 (47.9%)
Erythrocytosis	132 (30.5%)	118 (30.6%)	14 (29.2%)
Normal	84 (19.4%)	73 (19.0%)	11 (22.9%)

*Hematocrit*			
Low	271 (62.6%)	244 (63.4%)	27 (56.3%)
High	81 (18.7%)	70 (18.2%)	11 (22.9%)
Normal	81 (18.7%)	71 (18.4%)	10 (20.8%)

*Leukocyte*			
Leukopenia	124 (28.63%)	111 (89.5%)	13 (10.5%)
Leukocytosis	32 (7.39%)	29 (90.6%)	3 (9.4%)
Normal	277 (63.97%)	245 (88%)	32 (11.6%)

*Neutrophil*			
Neutropenia	11 (2.5%)	7 (63.6%)	4 (36.4%)
Neutrophilia	323 (74.6%)	288 (89%)	35 (10.8%)
Normal	99 (22.9%)	90 (90.9%)	9 (9%)

*Lymphocyte*			
Lymphopenia	341 (78.85%)	296 (86.8%)	45 (13.2%)
Lymphocytosis	38 (8.8%)	35 (92.1%)	3 (7.9%)
Normal	54 (12.5%)	48 (88.95%)	6 (11.1%)

## Data Availability

The data used to support the study are available from the corresponding author on request.
